# The Effect of Adding Different Elements (Mg, Fe, Cu, and Ce) on the Properties of NiCo_2_O_X_ for CO-Catalyzed Oxidation

**DOI:** 10.3390/ma18112554

**Published:** 2025-05-29

**Authors:** Jiefeng Wang, Zhili Chen, Tianqi Cao, Junsheng Yang, Yijian Kuang, Jiangang Kang

**Affiliations:** 1 School of Mechanical and Aeronautical Manufacturing Engineering, Anyang Institute of Technology, Anyang 455000, China; wangyufan2007@163.com; 2 College of Mechanical Engineering, Wuhan Polytechnic University, Wuhan 430048, China; caotianqi0328@163.com (T.C.); yangjunsheng2008@163.com (J.Y.); kuangyijian1999@163.com (Y.K.); 3 Zhongye Changtian International Engineering Co., Ltd., Changsha 410205, China; csukjg@126.com

**Keywords:** water and sulfur resistance, CO catalytic oxidation, cobalt- and nickel-based catalysts, adding different elements

## Abstract

In this experiment, NiCo_2_O_x_ catalysts, with different elements added (Mg, Fe, Cu, and Ce), were prepared using the co-precipitation method to investigate their catalytic performance for carbon monoxide, as well as their water resistance and sulfur resistance. Combined with the sintering flue gas environment of Baosteel Zhanjiang Iron and Steel Co., Ltd., it provides a reference for the catalytic oxidation of CO in complex environments. The results reveal that the Fe-added catalysts exhibited a better CO catalytic performance and possessed good redox properties, and the Fe metal ion-added NiCo_2_O_x_ catalysts showed a CO catalytic efficiency of 91.72% at 100 °C. Meanwhile, the Fe-added catalysts had the strongest resistance to water, with a conversion of 98.37% to CO at 140 °C, and with 10% water vapor. The Ce-added catalyst showed a better SO_2_ resistance and hybrid resistance of SO_2_ and H_2_O. Under the condition of sulfur addition, the CO conversion of the Ce-added catalyst was as high as 63.07% after 4 h of SO_2_ introduction, and the efficiency could be restored to 100% after cutting off the supply of SO_2_. Under the conditions of sulfur addition and water addition, the CO conversion of the catalyst was 98.23% after cutting off the SO_2_.

## 1. Introduction

CO is a colorless, odorless, and non-irritating toxic gas. When the volume fraction of CO in the environment is higher than 0.1%, it will cause a toxic reaction in the human body. CO is mainly derived from the incomplete combustion of fuels in the industrial production process, of which the iron and steel industry′s CO emissions are huge. Iron and steel production in China mainly relies on the long-process route, including sintering, pelletizing, coking, blast furnace smelting, converter steelmaking, and steel rolling [1]. Among these processes, the sintering process plays a crucial role in iron and steel production. However, the flue gas pollution problem is also the most prominent at this stage. The gaseous pollutants generated by this process account for nearly 40% of the total emissions in the iron and steel industry. The original emission concentration of CO in this flue gas is 800 times, 228 times, and 160 times that of the ultra-low emission concentrations of particulate matter, SO_2_, and NO_x_, respectively, and it is difficult to utilize the resources of this CO, which is generally directly discharged into the atmosphere to cause environmental pollution. In industrial production, CO is a toxic and regulated pollutant that corrodes equipment or poisons catalysts, leading to a significant reduction in productivity, making CO removal particularly important given the hazards of CO in industrial applications and the environment [2]. Studies have shown that catalytic oxidation technology is currently the most simple and practical method to eliminate CO pollution by oxidizing CO to non-toxic CO_2_ through catalysts. However, commercial catalysts for CO oxidation in exhaust gas purification are often active at high temperatures and easily poisoned at low temperatures [3], and low temperatures lead to CO molecules that are difficult to activate effectively. The efficiency of the conventional process of CO removal is generally less than 60%, so the development of low-temperature, highly efficient, and stable CO oxidation catalysts is of great importance.

Transition metal oxides have attracted significant attention from researchers due to their low cost, high catalytic activity, and abundance [4], among which cobalt- and nickel-based catalysts are considered good alternatives to precious metals. Co_3_O_4_ is a more active CO oxidation catalyst, which exhibits excellent activity and stability in low-temperature drying environments, and its crystal configuration is a spinel structure, with a molecular formula of AB_2_O_4_, which can be written as CoCo_2_O_4_. Dey [5] studied NiOx, Ni/Al_2_O_3_, Ni-Co-O, NiFeO_4_, and Ni/TiO_2_ nickel-based catalysts and showed that the most effective Ni/TiO_2_ catalysts achieved 100% conversion of CO at 50 °C, while NiOx and Ni_0.2_Co_0.8_ catalysts achieved full conversion at 145 °C and 120 °C, respectively. The least effective Ni/Al_2_O_3_ and NiFeO_4_ catalysts were fully converted at 365 °C and 280 °C, respectively.

Compared with the high cost of the precious metal deposition method and the complex preparation of the constructed shell–core-type molecular sieve method, ion addition is more suitable for industrial applications due to its low cost and convenient preparation characteristics. Jyoti et al. [6] used a co-precipitation method for the preparation of Ce-added modified Co/Al_2_O_3_, which was compared with the unmodified catalysts. The Ce addition promoted the distortion of the cobalt lattice structure, and this deformation led to an increase in the number of reactive oxygen species and oxygen vacancies. In addition, the Ce modification prevented the generation of the cobalt aluminate phase and promoted the generation of the CO-oxidized Co_3_O_4_ active phase. The Ce-added Co/Al_2_O_3_ catalysts exhibited the maximum H_2_O and SO_2_ tolerance due to the high density of oxygen vacancies. Su et al. [7] prepared transition metal (Co, Cu, Fe)-added CeO_2_ catalysts by the nanocasting method using three-dimensional mesoporous silica KIT-6 as a hard template. In the catalysts Co_3_O_4_, CuO, and Fe_2_O_3_, microcrystals were encapsulated by CeO_2_ nanocrystals, respectively, and a small amount of Co, Cu, and Fe ions were exposed on the surface and strongly interacted with CeO_2_. The interaction between these ions (Co, Cu, Fe) and Ce ions is maximized in the three-dimensional mesoporous structure, resulting in unique redox properties. The results show that the introduction of Co, Cu, and Fe can effectively increase the chemisorbed oxygen and oxygen vacancy concentration on the surface of metal-added CeO_2_ catalysts.

Combined with the analysis of the sintering flue gas environment at Baowu Zhanjiang Iron and Steel Co., Ltd. (Zhanjiang, China), the presence of SO_2_ and H_2_O is often accompanied in the CO catalytic process. Although elemental adding of nickel–cobalt-based catalysts has been reported to enhance the CO catalytic performance of the catalysts, there are few studies that consider the effects of SO_2_ and H_2_O on the catalytic performance of the catalysts. According to the existing technology, after the flue gas has been desulfurized and de-dusted, the content of particulate matter and alkali metals will be greatly reduced, but a small amount of SO_2_ (30 mg/m^3^) will still exist in the flue gas, in addition to the untreated water. These problems result in incomplete oxidation of the catalyst surface and the formation of carbon deposits, which can lead to a continuous decrease in catalyst activity and both of these can deactivate the catalyst by blocking the active sites or by the formation of stabilized sulfates [8], so a catalyst is needed that will maintain reliable performance under these practical conditions. In this paper, NiCo_2_O_x_ spinel catalysts added with various metal ions were prepared by the co-precipitation method. This method exhibits the advantages of a simple and convenient preparation process, low cost, and high production capacity, which makes it suitable for the requirements of large-scale industrial tail gas treatment. NiCo_2_O_x_ spinel catalysts have high CO conversion efficiency for CO at low temperatures [9] and good stability, but water and sulfur tolerance need to be further improved; therefore, ion adding was carried out on NiCo_2_O_4_ catalysts, and the NiCo_2_O_4_ catalysts were modified with the introduction of four metal ions, namely, Mg, Fe, Cu, and Ce, to study the effects of different metal ions on the CO conversion and sulfur resistance of NiCo_2_O_x_ spinel catalysts. X-ray diffraction (XRD), scanning electron microscopy (SEM), the Brunauer–Emmett–Teller (BET) method, H_2_ temperature-programmed reduction (H_2_-TPR), CO temperature-programmed desorption (CO-TPD), and in situ diffuse reflectance infrared Fourier transform spectroscopy (in situ DRFITS) were used to analyze the changes in CO conversion and water and sulfur resistance of NiCo_2_O_4_ catalysts before and after modification.

## 2. Experimental Method

### 2.1. Preparation of CO Oxidation Catalyst

Catalysts of different element-added Ni-Co composite oxides were prepared via the co-precipitation method: Ni:Co:X = 1:2:1 molar ratio (X is Mg, Fe, Cu, Ce, respectively) of C_4_H_6_NiO_4_·4H_2_O, C_4_H_6_CoO_4_·4H_2_O, and nitric acid X aqueous compounds were weighed and dissolved in 300 mL of deionized water to form solution A, and then placed into an ultrasonic cleaner for 30 min, equipped with anhydrous sodium carbonate solution B and sodium hydroxide solution C (2 mol/L) according to the same molar ratio (the sum of ionic concentrations of Ni and Co). Solution A was added drop by drop into solution B under the condition of water bath at 60 °C, and stirred with a magnetic stirrer at 600 r/min. During the process, Solution C was used to adjust the pH, and the pH of the solution was maintained at about 10 by controlling the amount of dropwise addition. At the end of the reaction, the stirring continued for 1 h, and the precipitate obtained was aged at 60 °C for 18 h. The precipitate was washed by filtration with deionized water until the filtrate was neutral. The obtained product was then heated and dried in a drying oven at 120 °C for 12 h. After cooling, the product was roasted in a muffle furnace at a heating rate of 5 °C/min to 600 °C for 6 h. Finally, different element-added NiCo_2_O_x_ composite oxides composite oxide catalysts were obtained, which were named Mg/NiCo_2_O_x_, Fe/NiCo_2_O_x_, Cu/NiCo_2_O_x_, and Ce/NiCo_2_O_x_, respectively.

### 2.2. Catalyst Activity Test

The CO removal performance was tested in an integrated catalyst activity evaluation unit at GASMET, Finland. The catalyst was loaded in a stainless steel reaction tube with a simulated flue gas volume of 1 L/min, an airspeed of 30,000 h^−1^ (GHSV), and gas components of 6000 ppm CO, 16% O_2_, and an equilibrium gas of N_2_. The gas concentration of the catalyst was gradually increased from 40 °C to 180 °C by means of an electrically heated furnace, with one temperature point taken at every 20 °C, and the gas components were allowed to remain at a steady state for another 10 min in each temperature point. The outlet CO concentration was monitored in real time by a portable FTIR multi-component gas analyzer, and the CO removal efficiency (%) was calculated using the following equation:

(1) $$\mathrm{CO}\;\mathrm{removal}\;\mathrm{efficient}=\frac{{\left[co\right]}_{in}-{\left[co\right]}_{out}}{{\left[co\right]}_{in}}\times 100\%$$

### 2.3. Characterization of Catalysts

The composition of the physical phases was tested using a D8 ADVANCE X-ray diffractometer (XRD) from Bruker, Karlsruhe, Germany, with a scanning range of 5–75°, a scanning speed of 5°/min, and a step size of 0.02. The agglomeration and sintering of the catalyst samples were observed using a Tescan Mira4 field emission scanning electron microscope (SEM) from Brno, Czech Republic. Programmed thermal reduction of H_2_ (H_2_-TPR) and programmed thermal desorption of CO (CO-TPD) were carried out using the Autochem II 2920, Micromeritics Instruments Corporation, Norcross, GA, USA. The species on the catalyst surface, as well as adsorbed species, were tested using a Nicolet is 20 Fourier transform infrared spectrometer (in situ DRIFTS) from Thermo Fisher Scientific Inc., Waltham, MA, USA. The above experimental tests were repeated three times.

## 3. Results and Discussion

### 3.1. XRD Characterization Results for Catalysts

**Figure 1:**
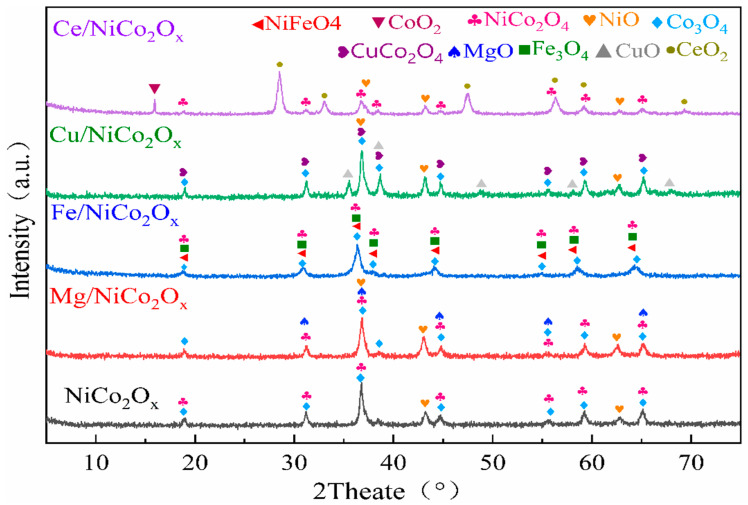
XRD patterns of NiCo_2_O_x_ catalysts added with different elements.

The phase constitution analysis of NiCo_2_O_x_ catalysts with different elements added is plotted in Figure 1. Unmodified NiCo_2_O_x_ catalysts with more NiCo_2_O_4_ and Co_3_O_4_ spinel in the phase and a small amount of NiO present; the NiCo_2_O_x_ composite oxide is added with Mg as the substrate, and some MgO is newly generated in the phase component, retaining NiCo_2_O_4_ and Co_3_O_4_ and a small amount of NiO; Fe-added NiCo_2_O_4_ catalysts, generating the corresponding Fe_3_O_4_ and a small amount of NiFeO_4_ oxides, Co_3_O_4_ is retained, stereotyped NiCo_2_O_4_ spinels are present, and dispersed Fe_3_O_4_ is not conducive to the formation of NiO; Cu-added NiCo_2_O_4_ catalyst, the newly generated CuO, CuCo_2_O_4_ also the presence of stereotyped Co_3_O_4_ spinel and a small amount of NiO, but NiCo_2_O₄ is absent. The Ce-added catalyst produces more CeO_2_ and NiCo_2_O_4_, and small amounts of NiO and CoO_2_ are also formed. The diffraction peaks at NiCo_2_O_4_ are brightly broadened, and it is believed that the crystals change from crystalline to amorphous. Table 1 shows the formation of phases with different element additions.

### 3.2. SEM Characterization of Catalysts

**Figure 2:**
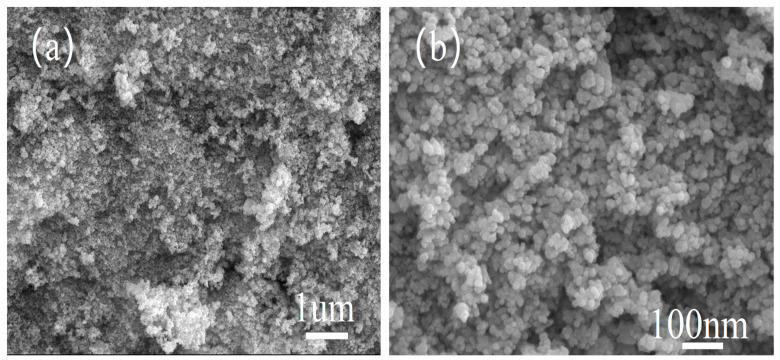
SEM patterns of NiCo_2_O_x_ catalyst. (**a**) 1 micron resolution (**b**) 100 nanometer resolution.

The NiCo_2_O_x_ composite oxide with the highest crystallinity was selected for SEM characterization, which is shown in Figure 2. From Figure 2a, it can be observed that the catalyst is distributed in the form of compact spherical clusters, and some of the particles are agglomerated, and the particles appear to be agglomerated mainly due to adsorption generated by intermolecular forces and electrostatic effects of nanoparticles [10]. From Figure 2b, it can be seen that the grains of NiCo_2_O_x_ samples are all in the nanometer size range, with dense grain arrangement, and are heterogeneous in size and shape, about 1–6 nm.

**Figure 3:**
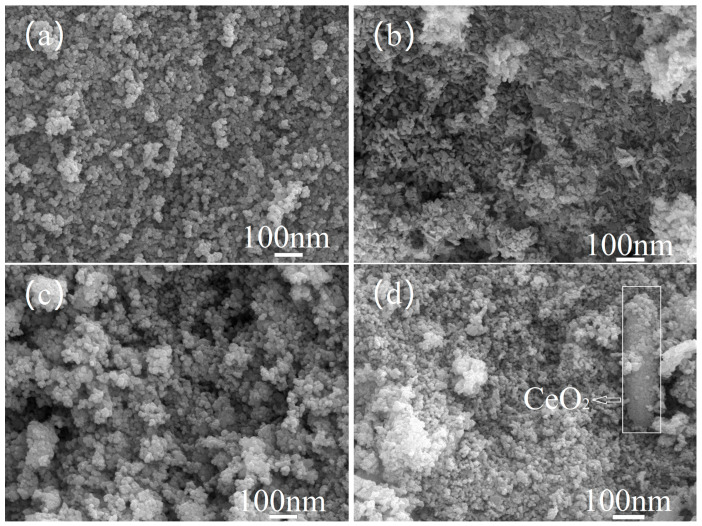
SEM spectra of NiCo_2_O_x_ catalysts added with different elements. (**a**) Mg/NiCo_2_O_x_, (**b**) Fe/NiCo_2_O_x_, (**c**) Cu/NiCo_2_O_x_, (**d**) Ce/NiCo_2_O_x_.

The surface morphology of NiCo_2_O_x_ composite oxide catalyst added with different elements is shown in Figure 3 below. It can be seen that the catalysts of NiCo_2_O_x_ added with Mg and Cu also show spherical clusters of agglomerated particles in the catalyst morphology as MgO and CuO themselves show spherical morphology characteristics; catalysts with Fe-added NiCo_2_O_x_ show a large number of nanowire-like morphologies inside, which are high-purity Fe_3_O_4_ crystals synthesized by the co-precipitation method [11]. The surface of the Ce-added NiCo_2_O_x_ catalyst was observed to have a rod-like structure with agglomerated particles adsorbed on it. The formation of these agglomerated particles was attributed to the adsorption of particles produced by NiCo_2_O_x_ on the rod-like CeO_2_ surface [12].

### 3.3. Catalyst Performance Evaluation

**Figure 4:**
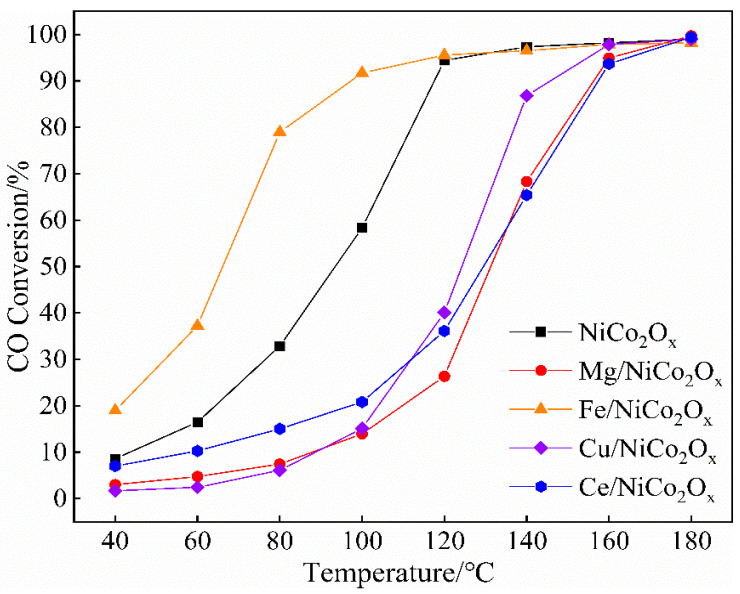
CO conversion on NiCo_2_O_x_ catalysts added with different elements.

Figure 4 shows the results of CO catalytic performance tests performed for the NiCo_2_O_x_ catalysts added with four metal ions: Mg, Fe, Cu, and Ce.

Analysis of the plots reveals that the conversion efficiency of CO increases as the catalytic temperature increases. The catalysts added with Mg, Cu, and Ce all showed a significant decrease in CO catalytic performance compared to the unmodified catalysts, while the effects of the adding of these three metal ions on the catalytic performance were not much different from each other. The Fe-added catalysts, however, showed significant performance enhancement. The CO catalytic efficiencies of NiCo_2_O_x_ catalysts with four metal ions added, namely Mg, Fe, Cu, and Ce, as well as unmodified catalysts were 14%, 91.7%, 15.1%, 20.8%, and 58.4%, respectively, at 100 °C. In particular, unmodified catalysts can achieve more than 90% CO conversion at 120 °C, while Mg- and Ce-added catalysts require up to 160 °C to achieve similar conversions.

### 3.4. BET Characterization of Catalysts

**Figure 5:**
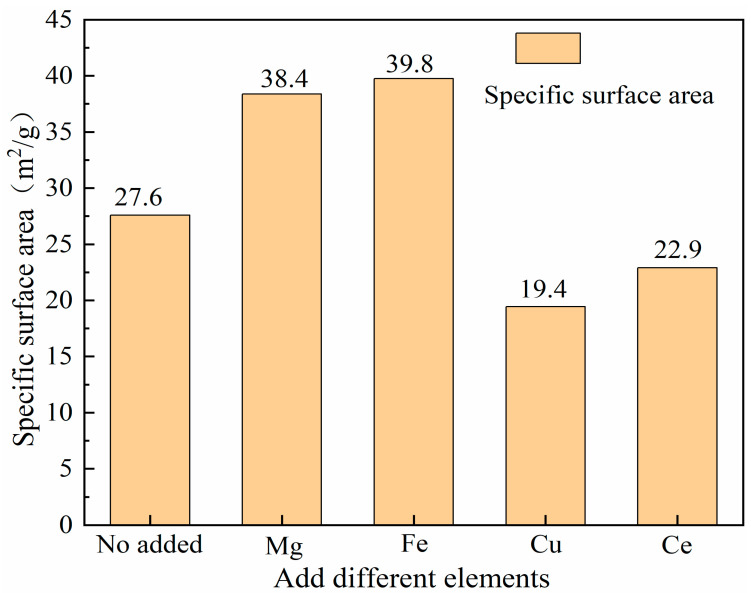
Specific surface area of NiCo_2_O_x_ catalysts with different elements added.

The specific surface areas of NiCo_2_O_x_ catalysts added with different elements and unmodified NiCo_2_O_x_ catalysts were characterized and compared, and the results are shown in Figure 5.

It can be seen from the figure that the catalyst added with Fe has the largest specific surface area. The specific surface areas of Mg- and Fe-added catalysts were 38.4 m^2^/g and 39.8 m^2^/g, respectively, compared with 27.6 m^2^/g for the unmodified catalysts, both of which were greatly improved, and the MgO-containing structure, the substitution of Mg^2+^, and the reduction in the particle size significantly increased the surface adsorption of oxygen in the Mg/NiCo_2_O_x_ catalysts [13], while the Fe_3_O_4_ nanowire morphology with abundant edge sites and crystalline surface exposure can facilitate the promotion of the contact between CO molecules and reactive oxygen species, improving the dispersion of Fe/NiCo_2_O_x_ catalysts, both of which are favorable to the increased specific surface area [14]. Although both have a relatively large specific surface area, the strong ionic bonding property of the MgO structure limits the lattice oxygen mobility, so its CO catalytic ability is not excellent, and the Fe-O bond is more prone to fracture and reorganization compared to the MgO bond, which supports rapid oxygen exchange. The specific surface areas of the Cu- and Ce-added catalysts were 19.44 m^2^/g and 22.91 m^2^/g, respectively, which both decreased to a certain extent compared with those of the unmodified catalysts, due to the high temperature calcination at 600 °C, which crystallized to produce large pieces of CuO and CeO_2_ particles [15], resulting in the decrease in surface area.

### 3.5. Characterization of Catalyst H_2_ Temperature Programmed Reduction (H_2_-TPR)

**Figure 6:**
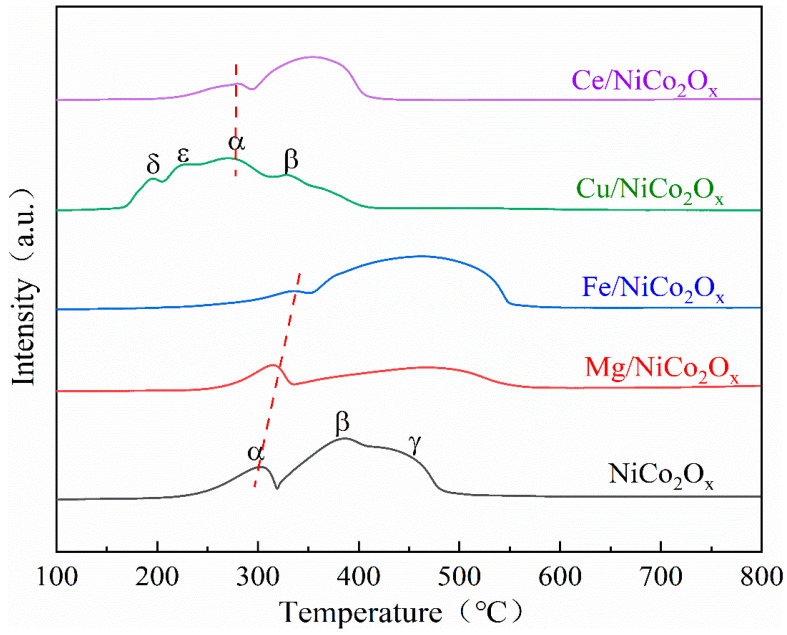
H_2_-TPR pattern of NiCo_2_O_x_ catalyst after modification.

H_2_-TPR was used to analyze the surface chemical reducibility of the elementally added NiCo_2_O_x_ catalysts, and the results are shown in Figure 6. As can be seen, the two reduction peaks of the Mg-added NiCo_2_O_x_ catalysts are shifted backward compared with the original sample, and the H_2_ consumption is greatly reduced, while the low-temperature catalytic activity is reduced [16]. The two reduction peaks of the Fe-added NiCo_2_O_x_ catalysts are also shifted backward compared with the original sample, and a wider reduction peak appears in the range of 300~550 °C due to the reduction of Fe_2_O_3_, which is carried out in a step-by-step manner (Fe_2_O_3_→Fe_3_O_4_→FeO). At the same time, the Co_3_O_4_ spinel is also reduced in this temperature range [17]. The Cu-added NiCo_2_O_x_ catalyst exhibits multiple reduction peaks, with the reduction peak at 195 °C attributed to CuO→Cu with less surface dispersion. The reduction peak at 225 °C is attributed to the larger surface bulk CuO→Cu or bulk CuO→Cu_2_O [18]. It can be seen that adding Cu leads to the reduction peaks of Co^3+^ and Ni^2+^ also becoming lower temperatures; the two reduction peaks of Ce-added NiCo_2_O_x_ catalysts are shifted to lower temperatures compared to the original samples.

Although the reduction peak of Fe-added NiCo_2_O_x_ catalyst shifted to the right, the area of its oxidation peak of 39.0179 was larger than the catalytic area formed by the other catalysts. The areas of Ce-, Cu-, Fe-, Mg-added and unmodified NiCo_2_O_x_ catalysts were 17.7, 31.23, 39.0, 23.5, and 38.8, respectively. The peak area is generally proportional to the number of active centers or reducible oxygen species on the catalyst surface. The Fe-added NiCo_2_O_x_ catalyst showed the highest H_2_ consumption, indicating the highest number of reducible active species and oxygen vacancies; the reduction of Fe^3^⁺→Fe^2^⁺ generates a large number of surface oxygen vacancies; and Fe^2^⁺ acts as an oxygen-buffering site by Fe^2^ ⁺ + O_2_→Fe^3^⁺-O, which improves the active site and is able to enhance its catalytic performance of CO [19]. Cu is easily oxidized to Cu⁺/Cu^2^⁺ in the reaction, which leads to the mismatch of the oxygen vacancy generation–disappearance rate and destroys the catalytic cycle, and CeO_2_ nanoparticles cover a part of the surface of Co_3_O_4_ to reduce the CO adsorption site, which are the reasons for the decrease in catalytic performance. Table 2 demonstrates the consumption of H_2_ after the addition of different elements.

### 3.6. Characterization of Catalyst with CO Temperature-Programmed Desorption (CO-TPD)

**Figure 7:**
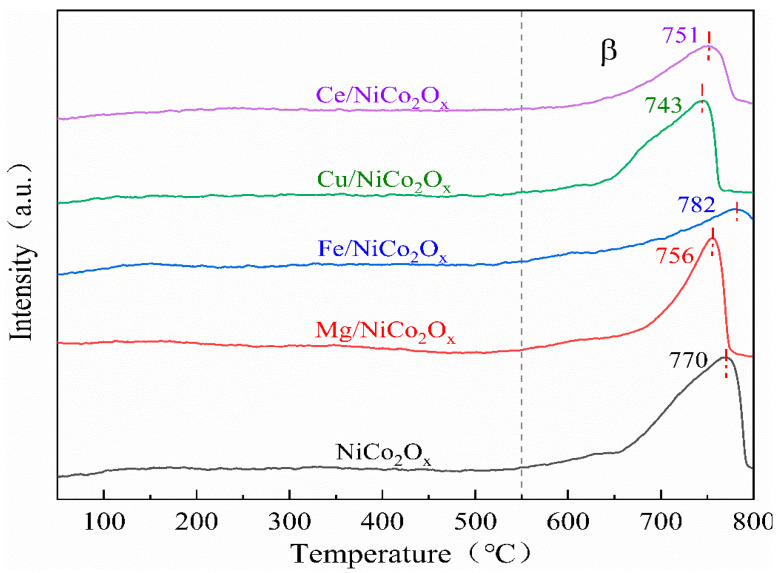
CO-TPD spectra of NiCo_2_O_x_ catalysts added with different elements.

To investigate the effect of elemental adding on the performance of NiCo_2_O_x_ catalyst in CO adsorption and resolution, it was characterized by CO-TPD, and the results are shown in Figure 7. It can be observed that all catalysts only have the regional peak of β. The β peak is the analytical peak for the formation of CO_2_ from CO and catalyst lattice oxygen. The temperatures of the highest peaks of the catalysts added with Mg, Cu, and Ce elements are all shifted to the left compared to the unmodified catalysts, while the highest peak of the catalysts added with Fe, the β peak, is gradually shifted to the direction of high temperature, The results show that the lattice oxygen content gradually increases with the addition of Fe, which also enhances the lattice oxygen mobility, and although the reaction requires higher temperatures, the abundance and circulation of lattice oxygen leads to the overall efficiency of the CO oxidation reaction. The high-temperature front suggests that the reaction pathway prefers the Mars–van Krevelen type mechanism, and CO adsorbs at oxygen vacancy sites and reacts with neighboring lattice oxygen, while O_2_ dissociates at Fe^3^⁺ sites to regenerate the oxygen vacancies [20], and more catalyst lattice oxygen is more favorable for the generation of CO_2_ with CO [21]. The CuO of Cu-added catalysts interacts with NiCo_2_O_4_ to produce a synergistic effect on CO oxidation, shifting the adsorption temperature to the left [22]; the CeO_2_ surface lattice oxygen of Ce-added catalysts also oxidizes CO [23], but the surface lattice oxygen has a limited oxygen storage capacity, and the low-temperature reaction occurs only locally, limiting the CO catalytic capacity.

### 3.7. In Situ Infrared Diffuse Reflection Characterization of Catalysts

**Figure 8:**
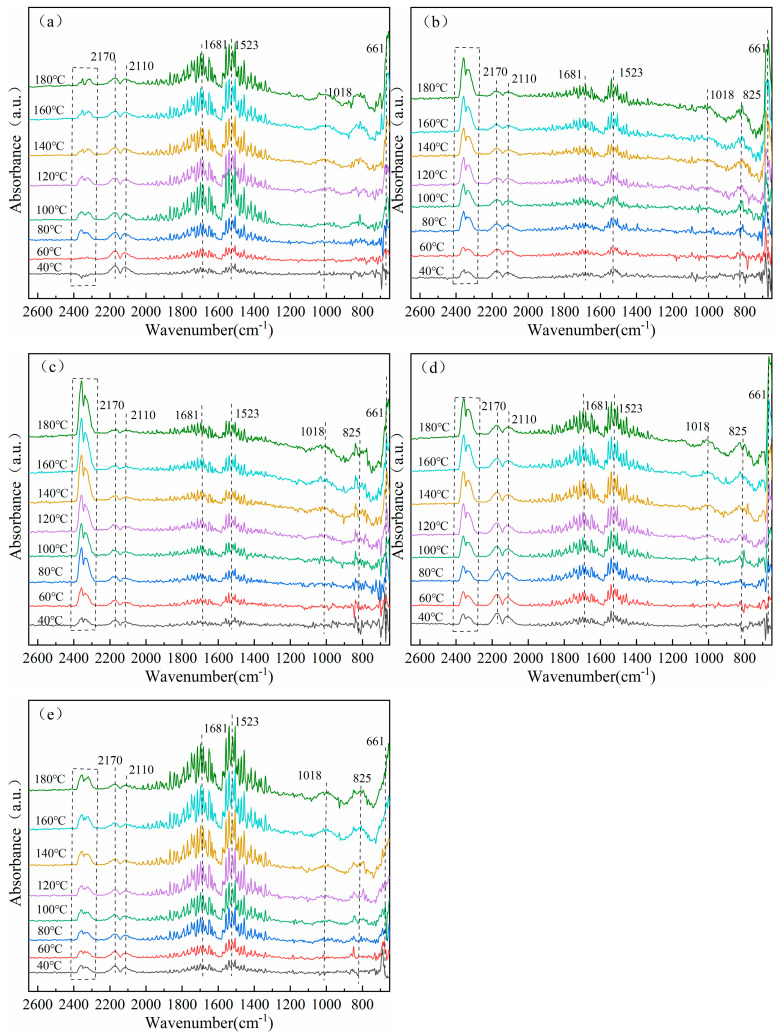
Adsorption of CO In situ DRIFTS on NiCo_2_Ox catalysts added with different elements. (**a**) NiCo_2_O_x_ (**b**) Mg/NiCo_2_O_x_ (**c**) Fe/NiCo_2_O_x_ (**d**) Cu/NiCo_2_O_x_, (**e**) Ce/NiCo_2_O_x_.

To investigate the surface reaction mechanism, in situ DRIFTS spectra of CO and O_2_ co-adsorption were obtained under simulated CO and O_2_ reaction conditions, as shown in Figure 8. Figure 8a shows the in situ DRIFTS plots of CO interactions at different temperatures for the NiCo_2_O_x_ catalyst. The absorption peaks at 1523 and 1681 cm^−1^ are assigned to monodentate carbonate species [24]. The vibration of adsorbed carboxylate (vs(COO-)) shown at 1018 cm^−1^ is highly stable [25]. The weaker diffraction peaks at 2110 and 2170 cm^−1^ are associated with the CO gas-phase concentration, attributed to the lower concentration in the feed gas [26]. Meanwhile, the peaks in the range of 2300–2400 cm^−1^ are attributed to gaseous CO_2_, which increase significantly with temperature increasing from 40 to 100 °C, and the gaseous CO_2_ peak intensity remains essentially unchanged after 100 °C due to the saturation of CO_2_ formation by CO combining with the surface reactive oxygen species [27]. In addition, there is a sharp peak at 661 cm^−1^ related to the Co^3+^-O stretching vibration [28]. For different elemental adding of the four samples, similar results were observed as in Figure 8a: bands of various carbonate species formed by surface adsorption of CO molecules also appeared in the range of 800–1700 cm^−1^ [29]. The peaks at 2110, 2170 cm^−1^, and 2300–2400 cm^−1^ are from gaseous CO and gaseous CO_2_, respectively.

It can be seen that the gaseous CO_2_ peaks of the different elementally added samples are enhanced with the increase in temperature, while the gaseous CO_2_ peaks of the Mg- and Fe-added catalysts in Figure 8b,c are stronger at 2300–2400 cm^−1^, which reflects that the CO adsorption on the surface of the Mg- and Fe-added catalysts is better under oxygen-rich conditions, The rapid temperature-dependent increase in the intensity of the CO_2_ peak at 2300–2400 cm^−1^ and the weaker carbonate peak (800–1700 cm^−1^) of the Fe-added catalysts indicate that CO prefers to react directly with lattice oxygen to form CO_2_ rather than forming intermediate state carbonates, while the gaseous CO_2_ peaks of the Cu- and Ce-added catalysts in Figure 8d,e are weaker, which is also consistent with the results of the catalyst evaluation tests. It is generally accepted that in Cu- and Ce-added catalysts, the CuO and CeO_2_ samples cause CO molecules to be oxidized by the surface active oxygen species to form a large number of carbonates, which are deposited on the surface of the catalysts, but only a few of each are converted to CO_2_ at low temperatures [30]. Therefore, these carbonates cover the active sites covering the catalyst surface that reduce the CO catalytic efficiency, resulting in the weaker gaseous CO_2_ peaks, while the gaseous CO peaks at 2110 and 2170 cm^−1^ and the carbonate peaks in the range of 800–1700 cm^−1^ are stronger.

## 4. Study on Water and Sulfur Resistance of NiCo_2_O_x_ Catalysts

After desulfurization of the flue gas from the iron and steel sintering flue gas, a large amount of water vapor remains, accounting for approximately 10% of the total gas content, along with trace amounts of SO_2_, with a concentration of 78.6 mg/m^3^. Due to the relatively low concentration of SO_2_ in the sintering flue gas, the catalyst deactivates slowly under such conditions. To rapidly verify the sulfur resistance performance of the catalyst, the SO_2_ concentration in this experiment was increased to 350 mg/m^3^. The CO catalytic performance of NiCo_2_O_x_ catalyst added with different elements was investigated, respectively, under three conditions: 10% H_2_O, 350 mg/m^3^ SO_2_, and a combination of 10% H_2_O and 350 mg/m^3^ SO_2_.

### 4.1. Performance of H_2_O Resistance

**Figure 9:**
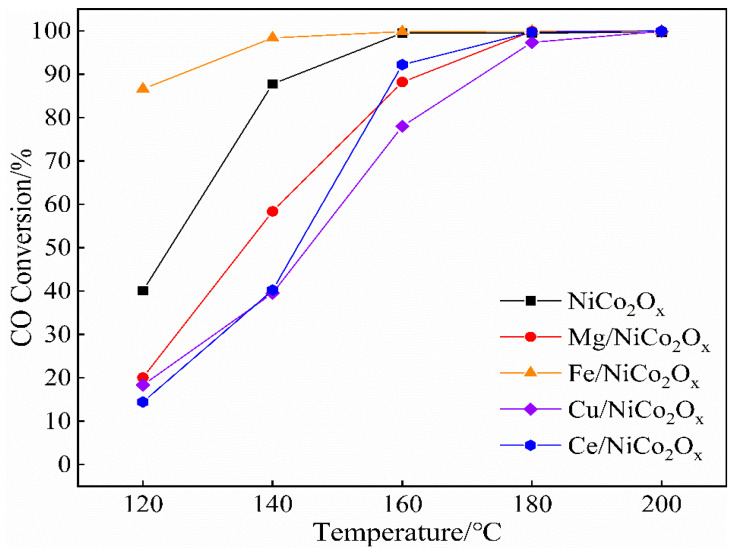
CO conversion of NiCo_2_O_x_ catalyst with different elements added at 10%H_2_O.

To investigate the effect of metal element adding on the water modification of NiCo_2_O_x_ catalyst, the results of simulated sintering flue gas test are shown in Figure 9.

From the figure, it can be observed that except for Fe addition which improved the water resistance of the catalyst, Mg, Cu, and Ce elements addition which all showed a decrease, and the catalytic efficiency of the catalyst with Fe added, which had the best water resistance, was 98.37% at 140 °C under the condition of containing 10% water vapor, when the catalytic efficiencies of the catalysts added with and unmodified by the elements of Mg, Cu, and Ce were only 58.4%, 39.5%, 40.2%, and 87.8%.

### 4.2. Performance of SO_2_ Resistance

**Figure 10:**
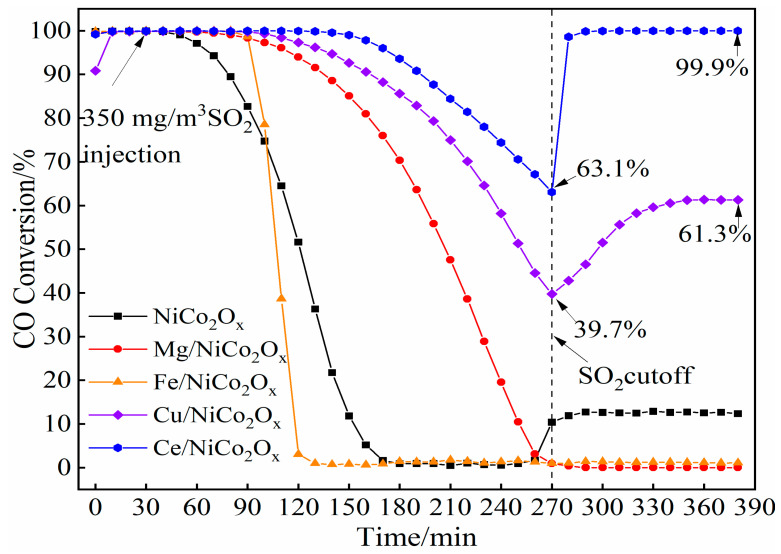
CO conversion of NiCo_2_O_x_ catalyst with different elements added at 200 °C and 350 mg/m^3^ SO_2_.

The modification results of adding different metal elements on the anti-sulfur performance of NiCo_2_O_x_ catalysts are shown in Figure 10. From the experimental results, it is obvious that the adding of metal elements has a significant effect on the anti-sulfur performance of NiCo_2_O_x_ catalyst. In particular, the Fe-added catalysts showed a decrease in sulfur resistance compared to the unmodified catalysts, while the Mg-added catalysts showed superior performance, with complete deactivation only after 90 min of SO_2_ exposure. The Cu- and Ce-added catalysts, on the other hand, exhibited significantly improved sulfur resistance, with the Cu-added catalysts maintaining 39.7% conversion after 4 h of to SO_2_ exposure, while the Ce-added catalysts achieved up to 63.1% conversion. After cutting off the SO_2_ supply, the Cu-added catalyst recovered to 61.3% efficiency, while the Ce-added catalyst fully recovered to 99.9% efficiency.

### 4.3. Performance of Hybrid SO_2_ and H_2_O

**Figure 11:**
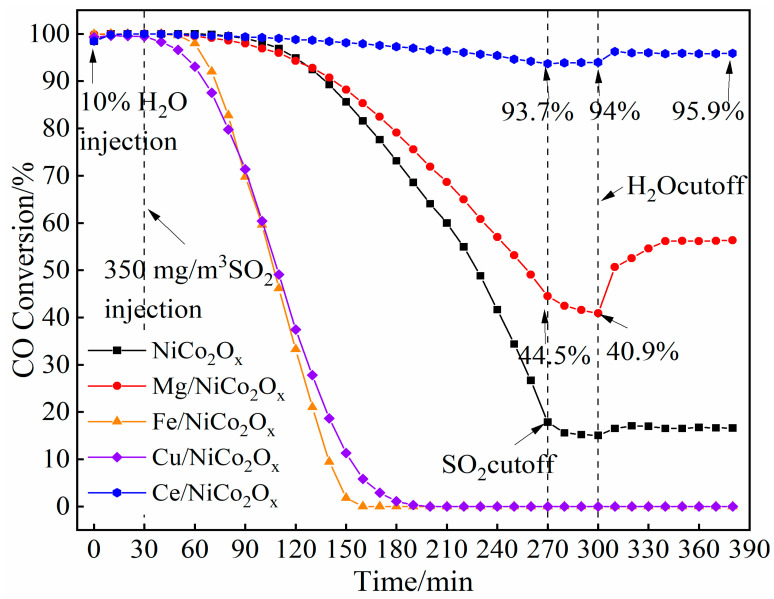
CO conversion of NiCo_2_O_x_ catalyst added with different elements at 200 °C, 10% H_2_O, and 350 mg/m^3^ SO_2_.

Figure 11 shows the simulated sintering flue gas test results of NiCo_2_O_x_ catalysts with the addition of different metal elements. From the figure, it can be observed that the extent of sulfur poisoning was retarded in the sulfur-plus-water condition for the unmodified NiCo_2_O_x_ catalyst compared to the sulfur-only condition. Under the condition of adding sulfur and water, the performance of Ce- and Mg-added NiCo_2_O_x_ catalysts was significantly improved, and there were still 94% and 40.9% CO conversions after cutting off SO_2_ for four hours of exposure to SO_2_ and 6 h of water, respectively, which could be recovered to 95.9% and 56.4%, respectively, after cutting off water. It can be seen that the Ce-added catalyst exhibits strong water and sulfur resistance, although as mentioned above, CeO_2_ reduces the CO catalysis, but under the condition of sulfur and water addition, CeO_2_ can enhance hydrophobicity to reduce the H_2_O adsorption and inhibit the formation of sulfate species, alleviating the inhibitory effects of H_2_O and SO_2_ on CO oxidation [31]. The performance of the Cu-added catalyst, on the contrary, decreased dramatically under the condition of adding sulfur and water, and it was essentially completely poisoned after 2 h. Under sulfur-rich, oxygen-rich, and water vapor conditions, H_2_O, SO_2_, and O_2_ react to form H_2_SO_4_ to acidify the catalyst [32], leading to the enhancement of the sulfur-resistant performance of the unmodified NiCo_2_O_x_ and Mg- and Ce-added catalysts, whereas the Cu- and Fe-added catalysts may be the direct generation of sulfate species such as CuSO_4_ and Fe_2_(SO_4_)_3_ leading to the performance decline.

The poisoning of Ce-added catalysts is reversible. Reversible poisoning means that the catalytic performance of the catalyst can be partially or completely restored when poisoning conditions are terminated; irreversible poisoning means that the catalyst loses its catalytic activity due to chemical or structural changes after the poisoning conditions are stopped. The reversible poisoning of Ce-added catalysts may be due to the fact that during sulfur poisoning, the SO_2_ adsorbed on the surface of the catalysts and Ce generated sulfate substances such as Ce_2_(SO_4_)_3_, which can be decomposed at low temperatures. Simultaneously, the structure of CeO_2_ prevents the further penetration of SO_2_.

## 5. Conclusions

(1) NiCo_2_O_x_ catalysts were modified by elemental addition. Compared with the undoped catalysts, the Mg-, Cu-, and Ce-doped catalysts all exhibited a significant decrease in CO catalytic performance. Notably, only the Fe-added low-temperature catalytic CO activity gained an enhancement, with a CO conversion of up to 91.7% at 100 °C. Under the oxygen-enriched conditions, the catalyst surface of Fe has good CO catalytic oxidation performance.
(2) Fe addition enhanced water resistance, achieving an efficiency of 98.4%. Under the same temperature and 10% water vapor condition, the catalytic oxidation of CO efficiency of the rest of the catalysts decreased to different degrees. The Ce-added NiCo_2_O_x_ catalyst exhibited anti-SO_2_ performance. The conversion of Ce-added catalyst reached 63.1% after four hours of SO_2_ exposure, and it was able to recover to 99.9% efficiency after cutting off the SO_2_ supply.
(3) Under the condition of adding sulfur and water, the anti-sulfur performance of all catalysts was increased, except for the Cu-added catalyst. The performance of Ce-added NiCo_2_O_x_ catalysts and was significantly improved, with 94% CO conversion after exposure to SO_2_ for four hours and H_2_O for six hours, and the catalytic rate could be restored to 95.9% after cutting off SO_2_ and water, respectively.

## Figures and Tables

**Table 1:** Phase formation of catalysts added with different elements.

Catalyzer	Phase Formation
Ce/NiCo_2_O_4_	NiCo_2_O_4_, NiO, CeO_2_, CoO_2_
Cu/NiCo_2_O_4_	NiO, Co_3_O_4_, CuO, CuCo_2_O_4_
Fe/NiCo_2_O_4_	NiCo_2_O_4_, Co_3_O_4_, Fe_3_O_4_, NiFeO_4_
Mg/NiCo_2_O_4_	NiCo_2_O_4_, NiO, Co_3_O_4_, MgO
NiCo_2_O_4_	NiCo_2_O_4_, NiO, Co_3_O_4_

**Table 2:** H_2_ consumption of different added elements.

Catalysts	Attribution and Temperature of Reduction Peak					H_2_ Consumption (mmol/g)
	α	β	γ	δ	ε	
	Co_3_O_4_→CoO	CoO→Co/ NiO→Ni/ Fe_2_O_3_→Fe_3_O_4_→FeO	CoO→Co	CuO→Cu	Bulk CuO→Cu Bulk CuO→Cu_2_O	
NiCo_2_O_x_	303	386	450	/	/	11.5
Mg/NiCo_2_O_x_	315	478	/	/	/	6.3
Fe/NiCo_2_O_x_	333	467	/	/	/	11.8
Cu/NiCo_2_O_x_	227	332	/	195	225	10.9
Ce/NiCo_2_O_x_	281	352	/	/	/	6.0

## Data Availability

The data presented in this study are available on request from the corresponding author. The data are not publicly available due to the fact that some of them involve the company’s experimental secrets, which may cause negative impacts on the enterprise.
